# Characterization of auxiliary iron–sulfur clusters in a radical *S*‐adenosylmethionine enzyme PqqE from *Methylobacterium extorquens* AM1

**DOI:** 10.1002/2211-5463.12314

**Published:** 2017-10-18

**Authors:** Natsaran Saichana, Katsuyuki Tanizawa, Hiroshi Ueno, Jiří Pechoušek, Petr Novák, Jitka Frébortová

**Affiliations:** ^1^ Centre of the Region Haná for Biotechnological and Agricultural Research Faculty of Science Palacký University Olomouc Czech Republic; ^2^ Comprehensive Research Institute for Food and Agriculture Faculty of Agriculture Ryukoku University Otsu Japan; ^3^ Regional Centre of Advanced Technologies and Materials Department of Experimental Physics Faculty of Science Palacký University Olomouc Czech Republic; ^4^ Present address: School of Science Mae Fah Luang University Chiang Rai Thailand

**Keywords:** [4Fe–4S] cluster, Mössbauer spectroscopy, pyrroloquinoline quinone, radical SAM enzyme

## Abstract

PqqE is a radical *S*‐adenosyl‐l‐methionine (SAM) enzyme that catalyzes the initial reaction of pyrroloquinoline quinone (PQQ) biosynthesis. PqqE belongs to the SPASM (subtilosin/PQQ/anaerobic sulfatase/mycofactocin maturating enzymes) subfamily of the radical SAM superfamily and contains multiple Fe**–**S clusters. To characterize the Fe**–**S clusters in PqqE from *Methylobacterium extorquens* AM1, Cys residues conserved in the N‐terminal signature motif (CX_3_ CX_2_C) and the C‐terminal seven‐cysteine motif (CX_9–15_ GX_4_ CX_*n*_ CX_2_ CX_5_ CX_3_ CX_*n*_C; *n *= an unspecified number) were individually or simultaneously mutated into Ser. Biochemical and Mössbauer spectral analyses of as‐purified and reconstituted mutant enzymes confirmed the presence of three Fe**–**S clusters in PqqE: one [4Fe**–**4S]^2+^ cluster at the N‐terminal region that is essential for the reductive homolytic cleavage of SAM into methionine and 5′‐deoxyadenosyl radical, and one each [4Fe**–**4S]^2+^ and [2Fe**–**2S]^2+^ auxiliary clusters in the C‐terminal SPASM domain, which are assumed to serve for electron transfer between the buried active site and the protein surface. The presence of [2Fe**–**2S]^2+^ cluster is a novel finding for radical SAM enzyme belonging to the SPASM subfamily. Moreover, we found uncommon ligation of the auxiliary [4Fe**–**4S]^2+^ cluster with sulfur atoms of three Cys residues and a carboxyl oxygen atom of a conserved Asp residue.

**Abbreviations**
5′dA: 5′‐deoxyadenosine
anSME: anaerobic sulfatase maturating enzyme
anSMEcpe: anSME from *Clostridium perfringens*
DTT: dithiothreitol
PQQ: pyrroloquinoline quinone
RS: radical SAM
SAM: *S*‐adenosyl‐l‐methionine
SPASM: subtilosin/PQQ/anaerobic sulfatase/mycofactocin maturating enzymes
WT: wild‐type

PqqE is a radical *S*‐adenosyl‐l‐methionine (SAM) enzyme that catalyzes the initial reaction of pyrroloquinoline quinone (PQQ) biosynthesis, that is, *de novo* carbon‐carbon bond formation between glutamate and tyrosine residues of a precursor peptide PqqA 1. The reaction is facilitated by a small protein PqqD 1, which forms a complex with PqqA 2.

As other members of the radical SAM superfamily of enzymes, PqqE contains a characteristic N‐terminal CX_3_CX_2_C motif that ligates three of the four iron atoms of [4Fe**–**4S] cluster. Binding of SAM to the remaining iron atom facilitates reductive cleavage of SAM to methionine and 5′‐deoxyadenosyl radical, which then in most cases generates a substrate radical by abstracting a hydrogen atom from the substrate 3. In the case of PqqE, a carbon radical is formed on the γ‐carbon of conserved glutamate of PqqA peptide. This subsequently reacts with the ring C3 carbon of conserved tyrosine forming a carbon‐carbon bond 1.

In addition to the conserved N‐terminal binding motif, PqqE contains a C‐terminal seven‐cysteine motif (CX_9–15_GX_4_CX_*n*_CX_2_CX_5_CX_3_CX_*n*_C; *n *= an unspecified number) in the domain designated SPASM (subtilosin/PQQ/anaerobic sulfatase/mycofactocin maturating enzymes) 4. The enzymes with the full SPASM domain generally bind additional two [4Fe**–**4S] clusters (termed auxiliary clusters) with proposed diverse functions such as substrate binding and positioning or electron transfer 5. A subgroup of proteins belonging to the SPASM subfamily contains a shortened version of the SPASM domain (named a Twitch domain 6), which lacks the CX_2_CX_5_CX_3_C motif of the SPASM domain and binds a single auxiliary Fe**–**S cluster 5. So far only crystal structure of a SPASM domain‐containing protein, anaerobic sulfatase maturating enzyme (anSME) from *Clostridium perfringens* (anSMEcpe), shows that both auxiliary clusters are fully ligated by cysteine residues, one of the ligating cysteine residues being present upstream of the SPASM domain 6. Available sequence and crystal structure data on the SPASM/Twitch domain‐containing proteins, however, indicate that some of them have an open coordination site for substrate binding on an auxiliary Fe**–**S cluster or use another amino acid residue to coordinate the cluster 7.

As we reported previously 8, PqqE possibly contains three Fe**–**S clusters consisting mainly of [4Fe**–**4S]^2+^ and [2Fe**–**2S]^2+^ forms. To identify the binding of Fe**–**S clusters in PqqE, conserved Cys residues in both the N‐terminal and C‐terminal signature motifs have been individually or simultaneously mutated into Ser. Biochemical and spectrophotometric analyses of mutant enzymes confirmed the presence of three Fe**–**S clusters in PqqE: a radical SAM [4Fe**–**4S]^2+^ cluster at the N‐terminal region indispensable for the reductive homolytic cleavage of SAM into methionine and 5′‐deoxyadenosyl radical, and two auxiliary clusters (designated Aux I and Aux II clusters), one each [2Fe**–**2S]^2+^ cluster (Aux I cluster) and [4Fe**–**4S]^2+^ cluster (Aux II cluster), in the C‐terminal SPASM domain. While mutation of Cys residues predicted to bind Aux II cluster resulted in expected loss of cluster accompanied with moderate decrease in SAM cleavage activity, mutation in the Aux I site resulted in marked reduction in protein stability, but not loss of the [2Fe**–**2S]^2+^ cluster and SAM cleavage activity. The structure model of PqqE created by homology alignment‐based structure modeling together with analysis of respective mutant enzyme suggests uncommon ligation of a carboxyl group of an Asp residue to an iron atom of the Aux II cluster.

## Results

### Dual iron isotope analysis

**Figure 1:**
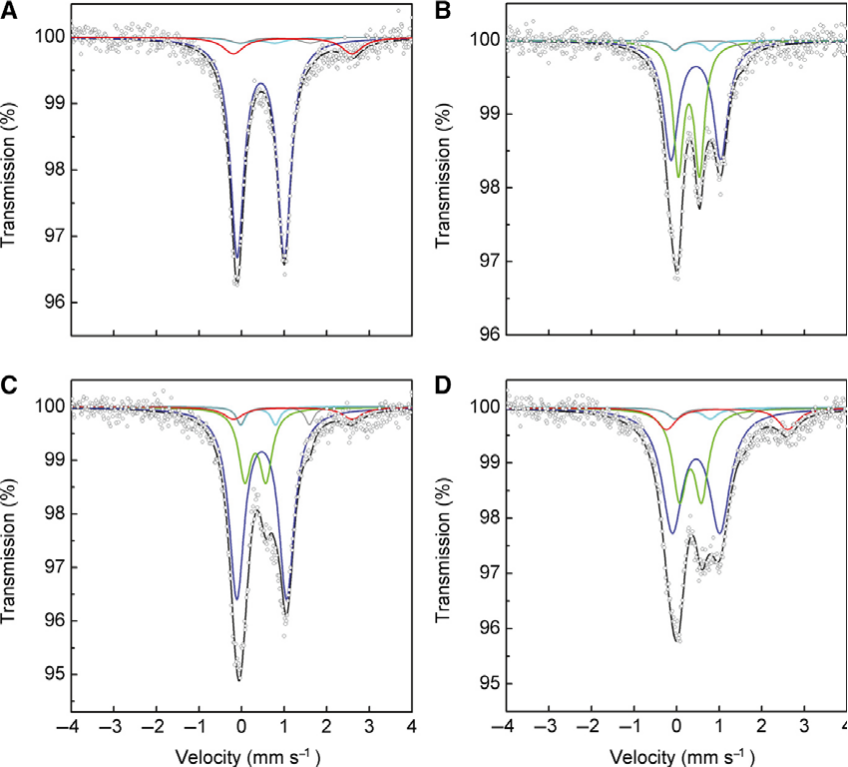
Mössbauer spectra of WT and C32S mutant of PqqE. ^57^Fe Mössbauer spectra of (A) ^56^Fe‐as‐purified WT enzyme reconstituted with ^57^Fe, (B) ^57^Fe‐as‐purified WT enzyme reconstituted with ^56^Fe, (C) ^57^Fe‐as‐purified WT enzyme reconstituted with ^57^Fe, and (D**)** ^57^Fe‐as‐purified C32S mutant reconstituted with ^57^Fe, all recorded at 5 K. Simulated spectra are shown as follows: blue, [4Fe–4S]^2+^; green, [2Fe–2S]^2+^; gray and light blue, ferrous and ferric sites, respectively, of the [4Fe–4S]^2+^ cluster ligated with the side‐chain carboxyl group of Asp319 at the Aux II site; red, free (noncluster) high‐spin Fe^2+^; black, the sum of the simulated spectra.

We have recently reported that the as‐purified and reconstituted enzymes of PqqE from *Methylobacterium extorquens* AM1 contain both [4Fe–4S]^2+^ and [2Fe–2S]^2+^ clusters as the major forms with the former being predominant in the reconstituted enzyme 8. In this study, we first employed a dual iron isotope (^56^Fe/^57^Fe) analysis to examine whether these Fe–S clusters are derived from biological cluster insertion by the *Escherichia coli* intracellular system or from chemical reconstitution or both. Thus, PqqE was initially expressed in *E. coli* cells in the presence of Mössbauer‐silent ^56^Fe^3+^ (natural abundance, 92%) and the ^56^Fe‐as‐purified enzyme was then reconstituted with ^57^Fe^3+^. Alternatively, PqqE was initially expressed in the presence of ^57^Fe^3+^ and the ^57^Fe‐as‐purified enzyme was then reconstituted with ^56^Fe^3+^. The ^56^Fe‐as‐purified PqqE reconstituted with ^57^Fe exhibited a rather simple Mössbauer spectrum (Fig. 1A) simulated with a single major quadrupole doublet (blue) assignable to a [4Fe–4S]^2+^ (*S*_total_ = 0) cluster (for Mössbauer parameters, see Table 1). In contrast, the ^57^Fe‐as‐purified PqqE reconstituted with ^56^Fe showed a complex spectrum with two major quadrupole doublets assignable to [4Fe–4S]^2+^ (*S*_total_ = 0) and [2Fe–2S]^2+^ (*S*_total_ = 0) clusters (Fig. 1B; blue and green, respectively), which resembles the complex spectrum of ^57^Fe‐as‐purified enzyme reported previously 8. This result indicates that reconstitution did not alter the ^57^Fe‐labeled cluster composition contained in the as‐purified enzyme. The spectra of ^57^Fe‐reconstituted enzymes (Fig. 1A,C) are accompanied with a small doublet (red) derived from the noncluster iron bound onto the protein surface with Mössbauer hyperfine parameters characteristic of free high‐spin Fe^2+^ ions (*S *=* *2) 9. In addition, the two minor unassigned doublets (gray and light blue) observed in all spectra have hyperfine parameters identical with those assigned to the [4Fe–4S]^2+^–pyruvate complex 10, as reported previously 9. They are assumed to be associated with ligation of the conserved Asp319 with an iron atom of the auxiliary [4Fe–4S]^2+^ cluster bound at the Aux II site, as described later. Using the values of relative spectral area (RA) of Mössbauer signals (Table 1), the [4Fe–4S]^2+^/[2Fe–2S]^2+^ cluster ratio is calculated to be approximately 0.75 [= {(53.1 + 3.4 × 2)/4}/(40.0/2)] and 1.9 [= {(67.1 + 3.6 × 2)/4}/(19.9/2)] in the ^57^Fe‐as‐purified enzymes reconstituted with ^56^Fe and ^57^Fe, respectively, with the minor doublets being included in the RA value of [4Fe–4S]^2+^. The cluster ratio in the as‐purified wild‐type (WT) enzyme (0.89; Table 2) was estimated to be slightly higher than that in the ^57^Fe‐as‐purified‐^56^Fe‐reconstituted enzyme. Thus, it is suggested that the cluster insertion into an expressed protein in *E. coli* cells may vary from cultivation to cultivation. Collectively, these results indicate that chemical reconstitution provides only the [4Fe–4S]^2+^ cluster and that the [2Fe–2S]^2+^ cluster contained in the as‐purified enzyme is not converted into the [4Fe–4S]^2+^ cluster by subsequent reconstitution. The Fe–S cluster bound at the consensus CX_3_CX_2_C motif located close to the N terminus of radical SAM enzymes is believed to be a [4Fe–4S]^2+^ cluster that is directly involved in the homolytic cleavage of SAM and hence often designated ‘RS cluster’ 3, 11, 12. Therefore, the [2Fe–2S]^2+^ cluster found in the as‐purified PqqE is probably bound at either the Aux I or Aux II site as defined below.

**Table 1:** Summary of the Mössbauer hyperfine parameters^a^

PqqE variant (Relating Fig.)	Color code for doublet component	δ ± 0.01 (mm·s^−^1)	*ΔE*_*Q*_ ±0.01 (mm·s^−1^)	RA ± 1 (%)	Cluster assignment^b^ spin *S*
WT					
^56^Fe‐as‐purified‐^57^Fe‐reconstituted (Fig. 1A)	Blue	0.45	1.10	83.6	[4Fe–4S]^2+^, *S *=* *0
	Light blue	0.38	0.82	3.2	[4Fe–4S]^2+^_Asp_, *S *=* *0
	Gray	0.78	1.62	3.2	[4Fe–4S]^2+^_Asp_, *S *=* *0
	Red	1.19	2.75	9.9	Free Fe^2+^, *S *=* *2
^57^Fe‐as‐purified‐^56^Fe‐reconstituted (Fig. 1B)	Green	0.30	0.49	40.0	[2Fe–2S]^2+^, *S *=* *0
	Blue	0.46	1.15	53.1	[4Fe–4S]^2+^, *S *=* *0
	Light blue	0.38	0.82	3.4	[4Fe–4S]^2+^_Asp_, *S *=* *0
	Gray	0.78	1.62	3.4	[4Fe–4S]^2+^_Asp_, *S *=* *0
^57^Fe‐as‐purified‐^57^Fe‐reconstituted (Fig. 1C)	Green	0.32	0.49	19.9	[2Fe–2S]^2+^, *S *=* *0
	Blue	0.47	1.17	67.1	[4Fe–4S]^2+^, *S *=* *0
	Light blue	0.38	0.81	3.6	[4Fe–4S]^2+^_Asp_, *S *=* *0
	Gray	0.78	1.62	3.6	[4Fe–4S]^2+^_Asp_, *S *=* *0
	Red	1.21	2.79	5.8	Free Fe^2+^, *S *=* *2
C32S					
^ 57^Fe‐as‐purified‐^57^Fe‐reconstituted (Fig. 1D)	Green	0.33	0.51	27.1	[2Fe–2S]^2+^, *S *=* *0
	Blue	0.46	1.10	55.9	[4Fe–4S]^2+^, *S *=* *0
	Light blue	0.38	0.82	3.6	[4Fe–4S]^2+^_Asp_, *S *=* *0
	Gray	0.79	1.62	3.6	[4Fe–4S]^2+^_Asp_, *S *=* *0
	Red	1.18	2.81	9.8	Free Fe^2+^, *S *=* *2
C323S					
^ 57^Fe‐as‐purified (Fig. 3B)	Green	0.32	0.49	57.8	[2Fe–2S]^2+^, *S *=* *0
	Blue	0.47	1.17	27.4	[4Fe–4S]^2+^, *S *=* *0
	Light blue	0.38	0.81	7.4	[4Fe–4S]^2+^_Asp_, *S *=* *0
	Gray	0.78	1.62	7.4	[4Fe–4S]^2+^_Asp_, *S *=* *0
^ 57^Fe‐as‐purified‐^57^Fe‐reconstituted (Fig. 3C)	Green	0.32	0.53	18.3	[2Fe–2S]^2+^, *S *=* *0
	Blue	0.44	1.12	65.5	[4Fe–4S]^2+^, *S *=* *0
	Light blue	0.39	0.82	2.4	[4Fe–4S]^2+^_Asp_, *S *=* *0
	Gray	0.78	1.62	2.4	[4Fe–4S]^2+^_Asp_, *S *=* *0
	Red	1.21	2.79	11.5	Free Fe^2+^, *S *=* *2
C310S/C313S					
^ 57^Fe‐as‐purified (Fig. 4B)	Green	0.28	0.54	70.7	[2Fe–2S]^2+^, *S *=* *0
	Blue	0.46	1.10	29.3	[4Fe–4S]^2+^, *S *=* *0
^ 57^Fe‐as‐purified‐^57^Fe‐reconstituted (Fig. 4C)	Green	0.32	0.49	19.3	[2Fe–2S]^2+^, *S *=* *0
	Blue	0.47	1.17	65.5	[4Fe–4S]^2+^, *S *=* *0
	Purple	1.42	1.32	5.0	Unknown
	Red	1.12	2.88	10.2	Free Fe^2+^, *S *=* *2
D319S					
^ 57^Fe‐as‐purified‐^57^Fe‐reconstituted (Fig. 5B)	Green	0.27	0.56	18.6	[2Fe–2S]^2+^, *S *=* *0
	Blue	0.46	1.12	70.8	[4Fe–4S]^2+^, *S *=* *0
	Purple	1.54	1.36	3.8	Unknown
	Red	1.25	2.72	6.7	Free Fe^2+^, *S *=* *2
C28S/C32S/C310S/C313S					
^ 57^Fe‐as‐purified (Fig. 6B)	Green	0.28	0.56	100.0	[2Fe–2S]^2+^, *S *=* *0
^ 57^Fe‐as‐purified‐^57^Fe‐reconstituted (Fig. 6C)	Green	0.28	0.56	26.5	[2Fe–2S]^2+^, *S *=* *0
	Blue	0.43	1.10	60.5	[4Fe–4S]^2+^, *S *=* *0
	Red	1.23	2.28	13.0	Free Fe^2+^, *S *=* *2

a δ is the isomer shift, Δ*E*_*Q*_ is the quadrupole splitting, and RA is the relative spectral area of individual components identified during spectral fitting, assuming an identical recoilless factor for all iron sites within the experimental error of Mössbauer technique.

b [4Fe–4S]^2+^_Asp_ represents an Fe atom of [4Fe–4S]^2+^ cluster interacting with the side‐chain carboxyl group of Asp319 at the Aux II site.

**Table 2:** Fe and S contents and SAM cleavage activities of the PqqE variants

PqqE variant	As‐purified				Reconstituted			
	Fe (mol·mol^−1^)	S (mol·mol^−1^)	Activity (nmol·min^−1^·mg^−1^)	Cluster ratio^a^	Fe (mol·mol^−1^)	S (mol·mol^−1^)	Activity (nmol·min^−1^·mg^−1^)	Cluster ratio^a^
WT	6.35 ± 0.62	10.15 ± 0.65	0.442 ± 0.012	0.89^b^	11.52 ± 1.05	16.17 ± 1.21	0.858 ± 0.071	1.9
C32S	3.84 ± 0.65^b^	5.30 ± 0.33^b^	nd^b^, ^c^	0.78^b^	10.50 ± 1.30^b^	12.10 ± 1.50^b^	nd^c^	1.2
C268S	2.12 ± 0.55	3.98 ± 0.93	0.021 ± 0.006	ND^c^	ND^c^	ND^d^	ND^d^	ND^d^
C323S	4.12 ± 0.57	7.29 ± 0.72	0.227 ± 0.066	0.37	10.34 ± 1.16	14.18 ± 1.72	0.868 ± 0.109	1.9
C313S	4.21 ± 0.54	7.52 ± 1.18	0.268 ± 0.022	ND^c^	10.36 ± 0.65	16.17 ± 0.76	0.469 ± 0.019	1.7
C310SC313S	4.09 ± 0.66	6.14 ± 0.81	0.322 ± 0.012	0.21	10.08 ± 1.12	13.58 ± 1.30	0.543 ± 0.042	1.7
C310SC313SC341S	3.72 ± 0.11	7.56 ± 0.42	0.253 ± 0.046	ND^c^	7.95 ± 0.80	12.01 ± 0.68	0.428 ± 0.108	ND^d^
D319S	6.15 ± 0.53	9.57 ± 0.78	0.410 ± 0.130	ND^c^	13.20 ± 1.70	17.89 ± 0.76	0.591 ± 0.031	1.9
C32SC310SC313S	1.69 ± 0.13	2.91 ± 0.17	nd^c^	0	7.41 ± 0.40	10.56 ± 1.46	nd^c^	0.9
C28SC32SC310SC313S	1.73 ± 0.17	2.90 ± 0.18	nd^c^	0	6.03 ± 0.28	9.51 ± 0.35	nd^c^	1.1
C28SC32SC310SC313SC341S	1.53 ± 0.12	3.10 ± 0.15	nd^c^	ND^c^	7.47 ± 0.50	11.34 ± 1.21	nd^c^	ND^d^

a Cluster molar ratio ([4Fe–4S]^2+^/[2Fe–2S]^2+^) was calculated from the relative area (RA) of Mössbauer signals including the RA of signals assigned to an Fe atom of [4Fe–4S]^2+^ cluster interacting with the side‐chain carboxyl group of Asp319 at the Aux II site (except for Aux II mutants: C313S, C310SC313S, and D319S).

b Data adopted from the previous study 8.

c Not detected.

d Not determined.

### Prediction of Fe–S cluster‐binding residues

Amino acid residues putatively involved in Fe–S cluster binding in PqqE have been predicted by homology alignment‐based structure modeling (SWISS‐MODEL) 13. Application of the entire amino acid sequence of *M. extorquens* AM1 PqqE (UniProt ID: P71517) as a query sequence resulted in building of three models with different template crystal structures: an anaerobic sulfatase maturating enzyme (anSME) from *C. perfringens* (anSMEcpe) (PDB ID: 4K36) 6, a molybdenum cofactor biosynthetic enzyme MoaA from *Staphylococcus aureus* (PDB ID: 1TV8) 14, and a maturase HydG involved in the [FeFe]‐hydrogenase H‐cluster assembly from *Thermoanaerobacter italicus* Ab9 (PDB ID: 4WCX) 15. Although all the enzymes autoselected as the template structures belong to the radical SAM superfamily, the first model with anSMEcpe as a template was employed in this study based on the highest GMQE (global model quality estimate) score (0.50) and overall sequence coverage (0.84). In addition, both PqqE and anSME belong to the SPASM subgroup of the radical SAM superfamily 4 and contain three Fe–S clusters, whereas MoaA and HydG both harbor only two Fe–S clusters. The initial model, however, had to be slightly modified in a partial region of PqqE (Ala247–Lys255) by adopting the coordinates of main chain atoms of the corresponding nonhomologous region of anSMEcpe (Ser254–Thr262), so that the cluster‐ligating Cys residues predicted for the Aux I site are placed at mutually nearby positions (see below). Finally, the coordinates of three [4Fe–4S]^2+^ clusters found in the anSMEcpe structure were merged into the PqqE model. The coordinates of a [2Fe–2S]^2+^ cluster taken from the structure of *E. coli* BioB 16 were also merged, instead of [4Fe–4S]^2+^, for the Aux I site.

**Figure 2:**
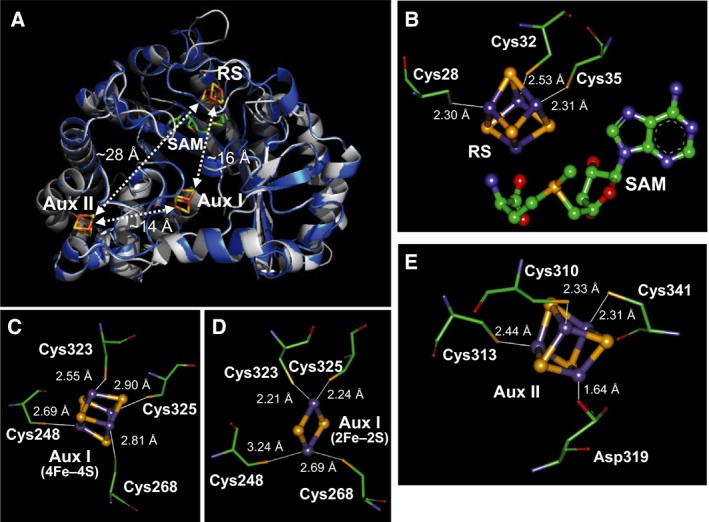
Homology alignment‐based structure model of PqqE. (A) The modeled PqqE structure is shown in ribbon representation (blue) overlaid with the template structure of anSMEcpe (gray) 6. The RS, Aux I, and Aux II clusters and SAM bound in anSMEcpe were merged into the modeled PqqE structure and are shown by stick models with respective atom color codes. Approximate distances between the clusters are also shown. (B–E) Enlarged views around the RS [4Fe–4S]^2+^ cluster and SAM (B), the Aux I site bound with a [4Fe–4S]^2+^ (C), or a [2Fe–2S]^2+^ cluster (D) and the Aux II [4Fe–4S]^2+^ cluster (E). Amino acid residues are shown in thin stick models and the clusters and SAM in thick ball‐and‐stick models. Chi angles (N‐CA‐CB‐SG) of Cys residues and that (N‐CA‐CB‐CG) of Asp319 were manually adjusted to have shortest possible distances (shown in Å) from Cys sulfur and Asp oxygen atoms to iron atoms (purple) of each cluster. In (C) and (D), the position and orientation of the clusters were also manipulated slightly within the cavity of the Aux I site.

The structure model of PqqE thus constructed (Fig. 2A) is consistent with the previous finding that most, if not all, radical SAM superfamily enzymes would have a common core fold comprising a partial (α/β)_6_ triosephosphate isomerase (TIM) barrel 17, 18. Three Cys residues (Cys28, Cys32, and Cys35) conserved in the consensus CX_3_CX_2_C motif are ideally located to bind the radical SAM [4Fe–4S]^2+^ cluster (RS cluster) (Fig. 2B) 3, 11, 12. Thus, manual adjustment of the chi angles (N‐CA‐CB‐SG) of Cys residues to ligate the RS cluster led the Cys sulfur‐to‐iron distances to 2.30–2.53 Å that are almost identical with those in the anSMEcpe structure (Fig. 2B). Furthermore, seven Cys residues conserved in the C‐terminal SPASM domain 8, 19 are positioned to bind two auxiliary Fe–S clusters at the sites almost identical with those identified in the anSMEcpe structure 6. The modeled Aux I site consists of four Cys residues (Cys248, Cys268, Cys323, and Cys325) and may be able to accommodate either [4Fe–4S]^2+^ (Fig. 2C) or [2Fe–2S]^2+^ (Fig. 2D), as detailed later. Cys248, Cys268, and Cys323 of PqqE correspond to Cys255, Cys276, and Cys330 of anSMEcpe, respectively; a Cys residue corresponding to Cys261 of anSMEcpe is not conserved in PqqE homologs 8 but the conserved Cys325 is instead placed in the Aux I site and may serve as a substitute for Cys261 of anSMEcpe (Fig. 2C,D). However, the Cys sulfur‐to‐iron distances could not be fully optimized even by rotating and/or slightly transferring the introduced Fe–S cluster within the cavity of Aux I site for both [4Fe–4S]^2+^ and [2Fe–2S]^2+^, which implies that the Aux I site was not modeled properly. In contrast, the Aux II site was modeled nicely to accommodate a [4Fe–4S]^2+^ cluster but was found to contain three Cys residues (Cys310, Cys313, and Cys341) (Fig. 2E) unlike in the anSMEcpe structure, in which the Aux II cluster is bound by four Cys residues (Cys317, Cys320, Cys326, and Cys348) 6. Cys310, Cys313, and Cys341 of PqqE correspond exactly to Cys317, Cys320, and Cys348 of anSMEcpe, respectively, with identical Cys sulfur‐to‐iron distances of 2.31–2.44 Å for the introduced [4Fe–4S]^2+^ cluster (Fig. 2E). Interestingly, Cys326 of anSMEcpe is replaced by an Asp residue (Asp319) in PqqE, which is totally conserved in PqqE homologs 8. As described later, the side‐chain carboxyl group of Asp319 is assumed to ligate an iron atom of the Aux II cluster. The modeled cluster distances (RS–Aux I, Aux I–Aux II, and RS–Aux II) are estimated to be about 16, 14, and 28 Å, respectively (Fig. 2A), which are comparable with those (16.9, 12.9, and 26.7 Å) in the anSMEcpe structure 6.

### Mutation of residues putatively involved in binding of RS and Aux I clusters

We previously mutated a Cys residue (Cys32) conserved in the consensus CX_3_CX_2_C motif and confirmed that the Cys32‐to‐Ser substitution leads to the complete loss of the reductive SAM cleavage activity associated with the RS cluster 8. The zero‐field ^57^Fe Mössbauer spectra of as‐purified (previous study) and reconstituted (present study, Fig. 1D) C32S mutants provided the [4Fe–4S]^2+^/[2Fe–2S]^2+^ cluster ratios of about 0.78 and 1.2 (Table 2), respectively; the cluster ratio (0.6) reported for the as‐purified C32S mutant 8 was recalculated by taking the two minor doublets into account. These results confirm the previous conclusion that the reconstituted C32S mutant contains no [4Fe–4S]^2+^ cluster at RS site but still retains [4Fe–4S]^2+^ and [2Fe–2S]^2+^ clusters at Aux I and II sites. The [2Fe–2S]^2+^ cluster is the more abundant form in both the as‐purified WT and C32S mutant enzymes 8.

To identify cluster‐binding Cys residues located at the Aux I site, we then mutated Cys268 and Cys323 individually into Ser and investigated the properties of the resultant mutants, C268S and C323S. Unfortunately, however, both C268S and C323S mutants were very unstable during anaerobic purification unlike the WT PqqE that is relatively stable even under semiaerobic conditions 8. Although the addition of 0.1 mm dithiothreitol (DTT) in the affinity purification buffer significantly improved the yield of soluble proteins of these mutants, chemical reconstitution with Fe^3+^ and S^2−^ in the presence of excess DTT resulted in further production of insoluble proteins, particularly for the C268S mutant. Thus, we could obtain only a small amount (~ 0.3 to 0.8 mg per 1 g of wet cells) of the as‐purified C268S mutant, which was insufficient for subsequent reconstitution and biochemical and spectral characterization. A moderate amount (~ 1.2 to 2.4 mg per 1 g of wet cells) of the as‐purified enzyme was obtained for the C323S mutant that could be used for further analysis.

Both as‐purified C268S and C323S mutants showed considerably lower SAM cleavage activities than the WT PqqE as summarized in Table 2. These results are consistent with the low Fe and S contents in the as‐purified mutant proteins and suggest that the Cys‐to‐Ser mutation at Aux I site also reduces the efficiency of [4Fe–4S]^2+^ cluster insertion into the RS site in *E. coli* cells. On the other hand, the reconstituted C323S mutant showed a high SAM cleavage activity comparable with that of the WT PqqE.

**Figure 3:**
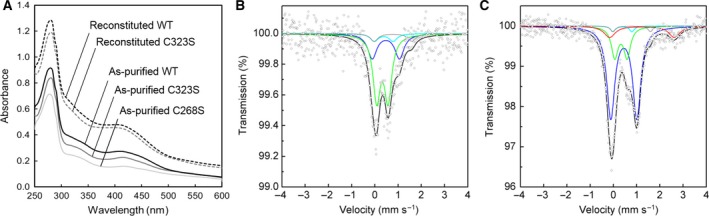
Spectral analyses of Aux I mutants of PqqE. (A) UV‐Vis spectra of the as‐purified and reconstituted C323S and C268S mutants and WT enzyme. (B, C) Mössbauer spectra of the as‐purified (B) and reconstituted (C) C323S mutant, recorded at 5 K. Simulated spectra are shown as follows: blue, [4Fe–4S]^2+^; green, [2Fe–2S]^2+^; gray and light blue, ferrous and ferric sites, respectively, of the [4Fe–4S]^2+^ cluster ligated with the side‐chain carboxyl group of Asp319 at the Aux II site; red, free (noncluster) high‐spin Fe^2+^; black, the sum of the simulated spectra.

As reported previously 8, the WT PqqE shows a UV‐Vis absorption spectrum that is typical for an Fe–S cluster‐containing protein with an absorption peak at 400–420 nm associated with the presence of [4Fe–4S]^2+^ clusters 20, 21 and shoulders at 300–350 nm and around 450 nm, which are derived from a [2Fe–2S]^2+^ cluster 20, 22 (Fig. 3A). As‐purified C268S and C323S mutants showed similar UV‐Vis spectra with considerably lower absorption bands throughout the measured wavelength region (250–600 nm) than the WT enzyme (Fig. 3A), in agreement with their low Fe and S contents (Table 2). The shoulder around 450 nm in the spectra of as‐purified C268S and C323S mutants is less visible than in the WT enzyme, presumably reflecting the difference in the contents of [4Fe–4S]^2+^ and [2Fe–2S]^2+^ clusters (see below). In contrast, the reconstituted C323S mutant exhibited a UV‐Vis spectrum comparable to the spectrum of the reconstituted WT enzyme (Fig. 3A).

Moreover, Mössbauer spectra of the as‐purified and reconstituted C323S mutants could not be simulated with a single quadrupole doublet (like the spectrum shown in Fig. 1A) but with a mixture of at least two major doublets. Although as‐purified C323S mutant showed a very noisy Mössbauer spectrum due to an inadequate protein amount as well as the low Fe content, the signals may be assignable to [4Fe–4S]^2+^ (*S*_total_ = 0) and [2Fe–2S]^2+^ (*S*_total_ = 0) clusters (Table 1 and Fig. 3B; blue and green, respectively). Similar parameters were obtained with the reconstituted C323S mutant that showed a less noisy spectrum (Table 1 and Fig. 3C; blue and green for [4Fe–4S]^2+^ and [2Fe–2S]^2+^ clusters, respectively). The [4Fe–4S]^2+^/[2Fe–2S]^2+^ cluster ratio was estimated to be about 0.37 [= {(27.4 + 7.4 × 2)/4}/(57.8/2)] in the as‐purified C323S mutant and 1.9 [= {(65.5 + 2.4 × 2)/4}/(18.3/2)] in the reconstituted one. Assuming that both RS and Aux II sites would bind only a [4Fe–4S]^2+^ cluster, as described above and below, we concluded that the C323S mutant can still bind [2Fe–2S]^2+^ cluster at the mutated Aux I site by utilizing the remaining three Cys residues (Cys248, Cys268, and Cys325; see Fig. 2C) with or without substituted Ser323.

### Mutation of residues putatively involved in binding of Aux II cluster

As described above, the predicted Aux II site consists of Cys310, Cys313, Cys341, and Asp319 (Fig. 2E). To investigate the involvement of the three Cys residues in binding of an Fe–S cluster at the Aux II site, we constructed single (C313S), double (C310S/C313S), and triple (C310S/C313S/C341S) Cys‐to‐Ser mutants and studied their properties. We also mutated Asp319 into Ser to examine the role of Asp319 in the Aux II cluster. In contrast to the Aux I mutants described above, all the Aux II mutants were efficiently expressed in *E. coli* cells and were as stable as the WT PqqE during anaerobic purification and subsequent reconstitution. As summarized in Table 2, iron and sulfur contents of the as‐purified and reconstituted Aux II single, double, and triple Cys‐to‐Ser mutants are similarly lower than those of the WT enzyme, indicating disruption of the Aux II site for binding of an Fe–S cluster. In contrast, even though the RS site of the reconstituted Aux II mutants is assumed to be fully occupied with a [4Fe–4S]^2+^ cluster, the SAM cleavage activities of all the reconstituted Aux II Cys‐to‐Ser mutants were only about a half (49–63%) of the reconstituted WT enzyme. These results show that the Fe–S cluster bound at the Aux II site ~ 29 Å apart from the RS cluster (Fig. 2A) indirectly affects the reductive SAM cleavage activity of the RS cluster.

**Figure 4:**
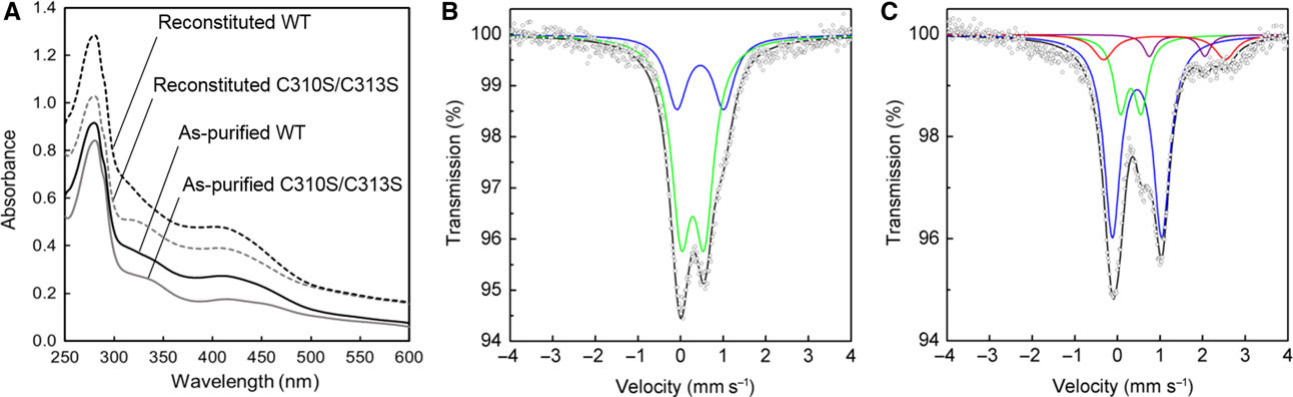
Spectral analyses of Aux II mutant of PqqE. (A) UV‐Vis spectra of the as‐purified and reconstituted C310S/C313S mutant and WT enzyme. (B, C) Mössbauer spectra of the as‐purified (B) and reconstituted (C) C310S/C313S mutant, recorded at 5 K. Simulated spectra are shown as follows: blue, [4Fe–4S]^2+^; green, [2Fe–2S]^2+^; red, free (noncluster) high‐spin Fe^2+^; purple, a doublet simulated for the unassigned signal; black, the sum of the simulated spectra.

As all the Aux II Cys‐to‐Ser mutants exhibited similar UV‐Vis and Mössbauer spectral properties, only those of the double mutant (C310S/C313S) are representatively shown in Fig. 4. As‐purified C310S/C313S mutant showed a UV‐Vis spectrum with a less intensive 400‐ to 420‐nm absorption peak than that of the as‐purified WT enzyme (Fig. 4A) and a Mössbauer spectrum showing a major quadrupole doublet assignable to [2Fe–2S]^2+^ (Fig. 4B, green). The [4Fe–4S]^2+^/[2Fe–2S]^2+^ cluster ratio is calculated to be 0.21 from the RA values shown in Table 1. On the other hand, reconstitution of the Aux II mutant (C310S/C313S) resulted in the production of the mutant enzyme having the cluster ratio of 1.7 (Table 2), which is inconsistent with the loss of Aux II cluster; the cluster ratio should be close to 1.0 (1 [4Fe–4S]^2+^ in the RS site and 1 [2Fe–2S]^2+^ in the Aux I site). A possible reason for apparent over‐reconstitution with a [4Fe–4S]^2+^ cluster is discussed later. The reconstituted Aux II mutant (C310S/C313S) exhibited another minor Mössbauer signal with an isomer shift (δ) of about 1.4 mm·s^−1^ (Fig. 4C, purple) that remains to be assigned.

**Figure 5:**
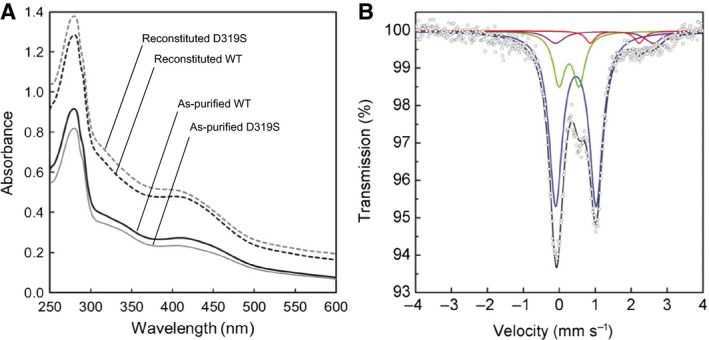
Spectral analyses of D319S mutant of PqqE. (A) UV‐Vis spectra of the as‐purified and reconstituted D319S mutant and WT enzyme. (B) Mössbauer spectra of the reconstituted D319S mutant, recorded at 5 K. Simulated spectra are shown as follows: blue, [4Fe–4S]^2+^; green, [2Fe–2S]^2+^; red, free (noncluster) high‐spin Fe^2+^; purple, a doublet simulated for the unassigned signal; black, the sum of the simulated spectra.

As identified in the structure model of PqqE (Fig. 2E), Asp319 has been predicted to ligate an Fe atom of the [4Fe–4S]^2+^ cluster bound at the Aux II site. Asp319 is conserved in all PqqE homologs 8. We inferred this uncommon ligation to be a source of the two minor unassigned doublets previously identified in the Mössbauer spectra of the reconstituted WT and C32S mutant enzymes 8. The doublets were also found in the reconstituted C323S mutant (Fig. 3C), but not in the Aux II mutant, C310S/C313S (Fig. 4C). Hyperfine parameters of these doublets are almost identical with those found in the [4Fe–4S]^2+^–pyruvate complex of 4‐demethylwyosine synthase from *Pyrococcus abyssi* 10, in which one of the four Fe atoms in a [4Fe–4S]^2+^ cluster has a more charge‐localized character of Fe^2+^ (showing wider doublets) than the delocalized Fe^2.5+^ upon binding of a cosubstrate pyruvate to an Fe atom of the [4Fe–4S]^2+^ cluster 23. The parameters for the ferrous site of a charge‐localized pair, Fe^2+^–Fe^3+^ (δ = 0.78 mm·s^−1^, Δ*E*_*Q*_ = 1.62 mm·s^−1^; Table 1), probably also correspond to those reported for the Asp ligand component (δ = 0.55 mm·s^−1^, Δ*E*_*Q*_ = 1.54 mm·s^−1^, measured at 77 K) of the oxygen sensing [4Fe–4S]^2+^ cluster of *Bacillus subtilis* transcriptional regulator Fnr 24. The reconstituted D319S mutant indeed showed a Mössbauer spectrum lacking the minor unassigned doublets, while giving the [4Fe–4S]^2+^/[2Fe–2S]^2+^ cluster ratio of about 1.9 (Fig. 5B), which is identical to that in the reconstituted WT enzyme (Fig. 1C). Its UV‐Vis spectrum (Fig. 5A) and Fe/S contents (Table 2) are also close to those of the WT enzyme. Altogether, these results strongly suggest that the side‐chain carboxyl group of the conserved Asp319 ligates an Fe atom of the [4Fe–4S]^2+^ cluster bound at the Aux II site but its mutation to Ser does not lead to the release of [4Fe–4S]^2+^ cluster from the Aux II site. Nevertheless, the reconstituted D319S mutant had a considerably decreased SAM cleavage activity comparable to those of other Aux II mutants. Thus, the ligation of the carboxyl group of Asp319 to the Aux II cluster is important for the indirect participation of the Aux II cluster in the SAM cleavage activity. The significance of Asp ligation for the presumed function of the Aux II cluster is discussed later.

### Mutation of multiple residues putatively involved in binding of RS and Aux II clusters

**Figure 6:**
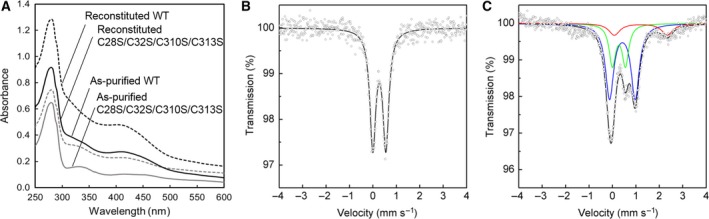
Spectral analyses of multiple RS/Aux II mutant of PqqE. (A) UV‐Vis spectra of the as‐purified and reconstituted C28S/C32S/C310S/C313S mutant and WT enzyme. (B, C) Mössbauer spectra of the as‐purified (B) and reconstituted (C) C28S/C32S/C310S/C313S mutant, recorded at 5 K. Simulated spectra are shown as follows: blue, [4Fe–4S]^2+^; green, [2Fe–2S]^2+^; red, free (noncluster) high‐spin Fe^2+^; black, the sum of the simulated spectra.

To further characterize the Fe–S cluster bound at the Aux I site, we finally mutated multiple Cys residues located at both RS and Aux II sites simultaneously into Ser (Cys28 and Cys32 at the RS site and Cys310, Cys313, and Cys341 at the Aux II site) and constructed a series of multiple mutants: C32S/C310S/C313S (designated triple mutant), C28S/C32S/C310S/C313S (quadruple mutant), and C28S/C32S/C310S/C313S/C341S (quintuple mutant), which are expected to bind only Aux I cluster. All these mutants were expressed efficiently in *E. coli* and could be purified in a sufficient amount for characterization. Because of the mutation at the RS site, all the three mutants showed no SAM cleavage activity both in the as‐purified and in reconstituted forms (Table 2). UV‐Vis spectral analyses revealed that all the as‐purified mutant enzymes show very low absorption in the wavelength region above 300 nm (Fig. 6A; only the spectra of quadruple mutant are shown), reflecting the considerable loss of Fe–S clusters. The absorption bands at 410 nm and 450 nm observed at almost the same intensity as well as the low Fe and S contents (Table 2) suggest the presence of only [2Fe–2S]^2+^ cluster in the as‐purified mutant enzymes, consistent with the Mössbauer spectrum of the as‐purified quadruple mutant (Fig. 6B) that exhibits only a single quadrupole doublet assignable to a [2Fe–2S]^2+^ cluster.

Surprisingly, however, chemical reconstitution of the multiply mutated enzymes resulted in marked increases in Fe and S contents (Table 2) and the absorption bands above 300 nm (Fig. 6A); if both the RS and Aux I sites were completely disrupted by the multiple mutation and the Aux I site was fully occupied with a [2Fe–2S]^2+^ cluster, then chemical reconstitution would not affect the Fe and S contents and UV‐Vis absorption spectra. Mössbauer spectrum of the reconstituted quadruple mutant (Fig. 6C) clearly revealed the presence of another quadrupole doublet (blue) assignable to [4Fe–4S]^2+^ cluster together with the small doublet of a nonspecifically bound Fe^2+^ ions (red). The [4Fe–4S]^2+^/[2Fe–2S]^2+^ cluster ratio in the reconstituted quadruple mutant is estimated to be about 1.1. Altogether, we conclude that only about 47% of the Aux I site of the as‐purified quadruple mutant is inserted with a [2Fe–2S]^2+^ cluster by the *E. coli* system, while the rest of the Aux I site remains vacant and can be reconstituted with a [4Fe–4S]^2+^ cluster. Thus, the Aux I site of PqqE has unique properties of being able to accommodate either [2Fe–2S]^2+^ (biologically) or [4Fe–4S]^2+^ (chemically) cluster. Furthermore, the cluster‐binding ability of the Aux I site is maintained even after the mutation of a single Cys residue contained in this site, as described above (see Fig. 3B,C).

## Discussion

The SPASM domain‐containing enzymes constitute the largest subfamily in the RS enzyme superfamily currently compiled in the structure–function linkage database 25; total number of functional domains registered in this subfamily is expanding to 16 026 as of July 2017. Many of these SPASM enzymes are involved in cofactor and peptide maturation processes, using ribosomally translated proteins (peptides), encoded near their genes, as substrate 4, 26. In addition to the RS [4Fe–4S]^2+^ cluster commonly bound to the N‐terminal signature motif (CX_3_CX_2_C), the SPASM enzymes have been reported to bind two auxiliary Fe–S clusters in the seven‐cysteine motif conserved in the C‐terminal half 5, except for those containing a partial SPASM motif (Twitch domain) that binds a single auxiliary cluster 7. As already described, the two auxiliary clusters in anSMEcpe have been both identified as [4Fe–4S]^2+^ clusters by X‐ray crystallography, in which the four iron atoms are fully ligated with four Cys residues in the seven‐cysteine motif plus one additional cysteine located outside the motif 6. A formylglycine‐generating enzyme AtsB, another member of the SPASM subfamily, contains two auxiliary [4Fe–4S]^2+^ clusters, one of which may be ligated only with three Cys residues 27. More recently, two other SPASM proteins, QhpD and AlbA involved in maturation of quinohemoprotein amine dehydrogenase 28 and synthesis of a 35‐residue, ribosomally synthesized bacteriocin subtilosin A 19, respectively, have also been predicted to contain two SPASM [4Fe–4S]^2+^ clusters coordinated by seven Cys residues.

As for PqqE, the enzyme from *Klebsiella pneumoniae* grown and isolated anaerobically was first reported to contain two [4Fe–4S]^2+^ clusters in its predominant form (probably one RS and another auxiliary ones) 29. However, we recently demonstrated that PqqE from *M. extorquens* AM1 contains most likely three Fe–S clusters consisting mainly of [4Fe–4S]^2+^ and [2Fe–2S]^2+^ forms 8. The contents of acid‐labile sulfide and iron and the extinction coefficient at 410 nm (about 42 000 m ^−1^·cm^−1^) of the reconstituted enzyme were consistent with this proposal as estimated from ε_410_ values reported for [4Fe–4S]^2+^ (~ 15 000 m ^−1^·cm^−1^) and [2Fe–2S]^2+^ (~ 8000–10 000 m ^−1^·cm^−1^) 22. Subsequently, the presence of two [4Fe–4S]^2+^ clusters and one in either [2Fe–2S]^2+^ or [3Fe–4S]^+^ states was also confirmed for *M. extorquens* AM1 PqqE by Barr *et al*. 1. Although the cuboidal [3Fe–4S]^+^ state has Mössbauer parameters similar to [2Fe–2S]^2+^ 23 and can be formed by gentle oxidation of [4Fe–4S]^2+^ (or back‐converted to [4Fe–4S]^2+^ by reconstitution with iron in the presence of DTT) 30, 31, we conclude that one of the Fe–S clusters contained in PqqE is [2Fe–2S]^2+^, based on the persistent Mössbauer signals that are not converted to those of [4Fe–4S]^2+^ by chemical reconstitution as described above. Thus, PqqE is the first example of SPASM proteins containing each one of [4Fe–4S]^2+^ and [2Fe–2S]^2+^ clusters at the auxiliary sites.

We then addressed the key issue of the present study: Which of the two predicted auxiliary sites (Aux I and II) binds which of [4Fe–4S]^2+^ and [2Fe–2S]^2+^ clusters? Although the final assignment was not as straightforward as we initially anticipated, we concluded that the Aux I site should bind a [2Fe–2S]^2+^ cluster and the Aux II site should bind a [4Fe–4S]^2+^ cluster under physiological conditions, based on the following observations. First (and most importantly), simultaneous mutation of multiple Cys residues predicted to be located at the RS and Aux II sites (such as triple and quadruple mutants; see Table 2) yielded the as‐purified mutant enzymes exhibiting only a single quadrupole doublet of Mössbauer signals (Fig. 6B), which is assignable to a [2Fe–2S]^2+^ cluster that should be bound at the unimpaired Aux I site. Second, all the Aux II mutants showed similar UV‐Vis and Mössbauer properties indicative of the presence of both [4Fe–4S]^2+^ and [2Fe–2S]^2+^ clusters (Fig. 4). As the RS site should bind only a [4Fe–4S]^2+^ cluster, this result strongly suggested that the Aux I site would bind a [2Fe–2S]^2+^ cluster. Third, three Cys residues (Cys310, Cys313, and Cys341) predicted to be located at the Aux II site by structure modeling are ideally situated to accommodate a cubic [4Fe–4S]^2+^ cluster (Fig. 2E), but not a rhombic [2Fe–2S]^2+^ cluster, similar to the three Cys residues (Cys28, Cys32, and Cys35) located at the RS site (Fig. 2B), whereas the Aux I site was modeled with some ambiguity for precise arrangement of the four predicted Cys residues (Cys248, Cys268, Cys323, and Cys325), suggesting a possibility to bind either [4Fe–4S]^2+^ (Fig. 2C) or [2Fe–2S]^2+^ (Fig. 2D). Consistent with the presence of four Cys residues at the Aux I site, most [2Fe–2S]^2+^ clusters have all cysteinyl (Cys)_4_ ligation with notable exceptions of (Cys)_2_(His)_2_ ligation in the Rieske ferredoxins 32, atypical (Cys)_3_(His)_1_ ligation in an outer mitochondrial membrane protein MitoNEET 33, 34, 35 and *E. coli* transcription factor IscR 36 and unique (Cys)_3_(Arg)_1_ ligation in biotin synthase (BioB) 16. Although it has been reported that the [2Fe–2S]^2+^ cluster can be reconstituted chemically into Fe–S proteins such as ferredoxins 37, dual iron isotope analyses indicated that the [2Fe–2S]^2+^ cluster is inserted into the Aux I site of PqqE only by the *E. coli* system(s).

Despite the cluster assignment as described above, we obtained contradictory results with the Cys‐to‐Ser mutant of the Aux I site (C323S), which clearly showed that the Aux I mutant did not lose the binding ability for a [2Fe–2S]^2+^ cluster (Fig. 3), and after reconstitution, it exhibited a high SAM cleavage activity comparable to the WT (Table 2). Consequently, we presumed that the mutated Aux I site may still bind a [2Fe–2S]^2+^ cluster utilizing the remaining three Cys residues (Cys248, Cys268, and Cys325). Alternatively (and more likely), the Ser residue substituting for Cys323 may participate in binding of a [2Fe–2S]^2+^ cluster together with the remaining three Cys residues. Such involvement of a Ser residue in ligating an iron atom of the [2Fe–2S]^2+^ cluster has been previously described for some Cys‐to‐Ser mutants of [2Fe–2S]^2+^ cluster‐containing proteins, though depending on the Cys residue to be substituted 38. To obtain a mutant without any Fe–S cluster at the Aux I site, mutation of multiple Cys residues may be needed. However, we did not attempt simultaneous mutation of two Cys residues predicted at the Aux I site due to the marked protein lability of even the single mutants (C268S and C323S).

Another unexpected observation relating to the Aux I site was that multiple Cys‐to‐Ser mutants of both the RS and Aux II sites (triple and quadruple mutants; see Table 2) were reconstituted with a [4Fe–4S]^2+^ cluster while retaining the [2Fe–2S]^2+^ cluster pre‐existing in the as‐purified enzymes (Fig. 6C), with the content of the [4Fe–4S]^2+^ cluster accounting for 53% in the reconstituted quadruple mutant. As the Aux I site in these mutants is the sole remaining cluster‐binding site and the [2Fe–2S]^2+^ cluster is not converted into a [4Fe–4S]^2+^ cluster by chemical reconstitution, as evidenced by dual iron isotope analysis, this result led us to assume that only about a half of the Aux I site is inserted with a [2Fe–2S]^2+^ cluster within *E. coli* cells and the remaining Aux I site is devoid of any cluster, which may be forced to accommodate a [4Fe–4S]^2+^ cluster by subsequent *in vitro* reconstitution, as implied by structure modeling (Fig. 2C). The assumption that a fraction of the Aux I site is vacant may also be applicable to the Aux II mutants, in which the [4Fe–4S]^2+^/[2Fe–2S]^2+^ cluster ratio significantly exceeds 1.0 after reconstitution; for example, the reconstituted single (C313S) and double (C310S/C313S) Aux II mutants had the cluster ratio of 1.7 (Table 2). It is thus likely that any mutation that leads to the loss of Aux II [4Fe–4S]^2+^ cluster affects the *in vivo* [2Fe–2S]^2+^ cluster insertion into the Aux I site, probably through conformational change in the overall protein folding, and the Aux I site remains consequently vacant in a fraction of the as‐purified mutant protein.

In marked contrast to the RS cluster that plays the well‐established common role for the reductive SAM cleavage, auxiliary clusters found in various SPASM/Twitch domain‐containing enzymes appear to have diverse functions, such as electron transfer, substrate anchoring, and substrate oxidation 5, 7, 39. Based on the crystal structure of anSMEcpe, one of the two auxiliary [4Fe–4S]^2+^ clusters, designated Aux I and located 16.9 Å from the RS cluster, has been proposed to function as an electron acceptor during substrate oxidation, while the second one (Aux II) located 12.9 Å from Aux I has been suggested to provide a route for the electron from the buried active site to the protein surface 6, 7. Similarly, the Aux I cluster of PqqE predicted to be located about 16 Å from the putative RS cluster (Fig. 2A) may accept an electron from a late radical intermediate derived from substrate PqqA, as proposed recently 1, although the Aux I cluster of PqqE inserted *in vivo* is not [4Fe–4S]^2+^ but [2Fe–2S]^2+^, which generally has higher redox potentials than the former 40. The predicted location of the Aux II cluster of PqqE is about 14 Å from the Aux I cluster and is closest to the protein surface (Fig. 2A), suggesting that the Aux II cluster serves for electron transfer between the Aux I cluster and an external electron acceptor. In view of the significantly reduced SAM cleavage activities of all the reconstituted Aux II mutants (Table 2), efficient electron transfer from the Aux II cluster to the RS cluster via the Aux I cluster may also be required even in the *in vitro* SAM cleavage reaction using dithionite as an electron donor, as proposed previously for RS enzymes carrying three [4Fe–4S]^2+^ clusters 19, 41. However, the possibility that the decreased activity of the Aux II mutants is due to the partial replacement of the Aux I [2Fe–2S]^2+^ cluster by [4Fe–4S]^2+^ or a consequence of perturbation of the redox or SAM‐binding properties of the RS cluster that are induced by loss of the Aux II cluster also cannot be excluded.

Finally, it is noteworthy that Asp ligation of Fe–S clusters is a rare example of non‐Cys ligands and has been found only for [4Fe–4S]^2+^ clusters 40, with the first identification in *Pyrococcus furiosus* ferredoxin 42 followed by *Desulfovibrio africanus* ferredoxin III 43, the dark‐operative photochlorophyllide reductase complex from *Rhodobacter capsulatus* 44, and the bacterial transcriptional regulator Fnr 24. In the photochlorophyllide reductase, Asp ligation was proposed to contribute to the low redox potential necessary to reduce photochlorophyllide 45. Although the reconstituted D319S mutant of PqqE did not lose the Aux II [4Fe–4S]^2+^ cluster, its SAM cleavage activity was considerably decreased (Table 2), suggesting that Asp ligation fine‐tunes the redox potential of the Aux II cluster to suit for the efficient electron transfer to the RS cluster catalyzing reductive SAM cleavage.

## Materials and methods

### Chemicals

*S*‐adenosyl‐l‐methionine, DTT, FeCl_3_, and LB broth were purchased from Sigma Aldrich (Munich, Germany). Li_2_S, ammonium iron (III) citrate, and ^57^Fe_2_O_3_ (96.64% isotopic enrichment) were purchased from Alfa Aesar (Karlsruhe, Germany), Lach‐Ner (Neratovice, Czech Republic), and ISOFLEX (San Francisco, CA, USA), respectively.

### Homology alignment‐based structure modeling

The structure model of PqqE was built by applying the *M. extorquens* AM1 PqqE amino acid sequence (UniProt ID: P71517) to the web site SWISS‐MODEL 13, and the resultant initial model was modified manually by Coot 46. The coordinates of [4Fe–4S]^2+^ and [2Fe–2S]^2+^ clusters were taken from those in the crystal structures of anSMEcpe (PDB ID: 4K36) [AdoMet (RS), Aux I and Aux II clusters] 6 and biotin synthase from *E. coli* (BioB) (PDB ID: 1R30) 16, respectively, and merged with the coordinates of PqqE model. The cluster–ligand distances were optimized manually using commercial software Discovery Studio (BIOVIA, Tokyo, Japan).

### Site‐directed mutagenesis

The C268S and C313S mutants of PqqE were prepared by PCR using Tks *Gflex*™ DNA polymerase (Takara, Tokyo, Japan) with phosphorylated forward (F) and reverse (R) primers containing a mismatched nucleotide listed in Table S1. The plasmid pET‐pqqE‐N prepared previously 8 was used as a template. The linearized PCR products were self‐ligated with T4 DNA Ligase (NEB, Ipswich, MA, USA) to yield pET‐C268S and pET‐C313S for the C268S and C313S mutants, respectively. The ligation mixtures were directly transformed into *E. coli* DH5α, and mutations were confirmed by DNA sequencing. For constructions of other PqqE mutants, site‐directed mutagenesis was carried out following a *Dpn*I‐mediated site‐directed mutagenesis method 47. The mutagenesis reactions were performed using a QuickChange Mutagenesis Kit (Agilent Technologies, CA, USA) following the manufacturer's recommended protocol. For constructions of the pET‐C310S, pET‐D319C, pET‐D319S, and pET‐C323S mutant plasmids, pET‐PqqE‐N was used as a template for PCR, and for construction of the pET‐C310S/C313S mutant plasmid, pET‐C313S was used. The primer pairs used in these reactions are listed in Table S1. Construction of pET‐C32S/C310S/C313S was carried out by digestion of pET‐C310S/C313S and pET‐C32S 8 with *Afl*II and *Bam*HI followed by ligation of the smaller fragment from pET‐C310S/C313S with the larger fragment from pET‐C32S. Construction of pET‐C28S/C32S/C310S/C313S was also performed using pET‐C32S/C310S/C313S as a template. The mutant plasmids were transformed into *E. coli* TOP10, and the mutations were confirmed by DNA sequencing. All mutant proteins thus constructed have an N‐terminal His_6_‐tag for purification with an affinity column as described below.

### Expression and purification of PqqE

*Escherichia coli* Rosetta 2 (DE3) cells (Novagen, Cambridge, MA, USA) harboring an appropriate plasmid were inoculated from a stock culture into 50 mL of LB medium containing kanamycin (50 μg·mL^−1^) and chloramphenicol (35 μg·mL^−1^) and cultivated overnight at 37 °C and 150 rpm. Overnight culture was inoculated (10 mL per 100 mL) into a fresh LB medium containing antibiotics and cultivated for 4 h at the same conditions. Then, 50 mL of this culture was transferred into 500 mL of fresh LB medium supplemented with antibiotics in one‐liter flasks, and the cells were grown at 37 °C and 150 rpm until reaching the OD_600_ of 0.9. The medium was supplemented with 0.1 mm of ^56^Fe^3+^ or ^57^Fe^3+^ and expression was induced by 0.1 mm isopropyl‐β‐d‐thiogalactopyranoside. Cells were further cultivated at 18 °C and 120 rpm for 16–18 h, harvested by centrifugation at 5700 ***g*** for 5 min, washed with distilled water bubbled with N_2_, and stored at −20 °C until use.

Purification of all PqqE variants was performed as previously described 8. All purification steps, except centrifugation, were carried out at room temperature (22 °C) in a glove box (SICCO, Grünsfeld, Germany) filled with nitrogen gas (O_2_ content 1.5 ppm) using buffer solutions that were bubbled with nitrogen for at least 30 min inside the glove box. The oxygen level inside the box was monitored with a GMH 3691 Digital Oximeter (GHM Messtechnik GmbH, Regenstauf, Germany). Cells suspended in the deoxygenated equilibration buffer (50 mm sodium phosphate, pH 8.0, 500 mm NaCl, 10 mm imidazole, and 10% (v/v) glycerol; 4.5 mL per 1 g of cell paste) were disrupted inside the glove box using 30‐minute incubation with BugBuster extraction reagent (0.5 mL per 1 g of cell paste) supplemented with 2.5 μL of benzonase per 1 g of cell paste (Novagen, Cambridge, MA, USA). The cell lysate was clarified by centrifugation at 16 700 ***g*** for 15 min at 4 °C in centrifugation tubes that were sealed inside the box. Supernatant was applied onto a Protino^®^ Ni‐NTA Agarose affinity column (Macherey‐Nagel, Düren, Germany) with a column volume of 5 mL, which had been pre‐equilibrated with the deoxygenated equilibration buffer. The column was washed with two volumes of the washing buffer (50 mm sodium phosphate, pH 8.0, 500 mm NaCl, 50 mm imidazole, and 10% (v/v) glycerol) and PqqE was then eluted with the elution buffer (50 mm sodium phosphate, pH 8.0, 500 mm NaCl, 135 mm imidazole, and 10% (v/v) glycerol). The buffer was then exchanged with buffer A (50 mm Tris/HCl, pH 8.0, 150 mm NaCl, 1 mm DTT, and 10% (v/v) glycerol) using a Sephadex G‐25 column (10 mL) and the as‐purified PqqE was immediately used for the subsequent experiments in most cases. Final yields of the as‐purified WT and mutant enzymes of PqqE, except for the mutants of Aux I site (C268S and C323S), were generally 5–6 mg per 1 g of wet cells. For Mössbauer spectral measurement, the purified protein was concentrated to about 0.5 mL in a sealed Amicon Ultra‐15 Centrifugal Filter Unit with 10‐kDa cutoff (Millipore, Darmstadt, Germany), frozen in liquid nitrogen inside the box, and stored in a sealed nitrogen‐filled bottle at −80 °C if not measured immediately. Typical concentration of PqqE for recording Mössbauer spectra was between 1.0 and 1.5 mm.

### Protein and activity assay

Soluble protein concentration was measured by the Bradford method 48 with a Bradford Protein Assay Kit (Bio‐Rad, Hercules, CA, USA) and bovine serum albumin as the standard. The values thus obtained were recalculated using Bradford correction factor of 1.025 8.

Assays of PqqE for the reductive homolytic cleavage of SAM were carried out at a room temperature (22 °C) in the anaerobic glove box using buffers bubbled with nitrogen as described 8. The Fe–S clusters of PqqE (2.5 mg·mL^−1^ in buffer A) were reduced by the addition of sodium dithionite (freshly prepared 1 m solution in buffer A) to the final concentration of 25 mm for 10 min. The enzyme reaction was started by the addition of SAM to the final concentration of 200 μm and was stopped after 30 min by heating at 80 °C for 10 min. The protein precipitates were removed by centrifugation at 17 500 ***g*** for 5 min, and the supernatant was analyzed for the formation of 5′‐deoxyadenosine (5′dA) using HPLC with UV detection at 259 nm. Reaction product, 5′dA, was analyzed on a Symmetry C18 column (2.1 × 150 mm, 5 μm; Waters, Milford, CT, USA) connected to an Alliance e2695 high‐performance liquid chromatograph (Waters) and a Waters 2998 photodiode array detector using a linear gradient of 15 mm ammonium formate, pH 4.0, and methanol 8. The concentration of product was calculated by a calibration curve method using authentic standard compound.

### Iron and sulfur quantification

Iron and acid‐labile sulfide contents were measured according to the methods of Fish 49 and Beinert 50, respectively.

### Reconstitution of PqqE

Reconstitution of Fe–S clusters was carried out under anaerobic conditions inside a glove bag (Sigma Aldrich, Munich, Germany) at 4 °C following the described procedure 8, 51. Briefly, PqqE (140 μm) reduced with 100 equivalents of DTT for 1 h was reconstituted by the addition of 10 equivalents of Fe^3+^ (in the form of ammonium iron citrate) followed, after five‐minute incubation with gentle stirring, by the addition of 10 equivalents of Li_2_S. After overnight incubation with gentle stirring and precipitate removal by centrifugation at 17 500 ***g***, the enzyme solution was purified under anaerobic conditions in a glove box on a Sephadex G‐25 column (10 mL) equilibrated with buffer A. For Mössbauer spectra measurement, the purified protein was concentrated and stored as described above.

### Spectral analyses of PqqE

UV‐Vis absorption spectra of anaerobic solutions of PqqE variants were measured in a sealed cuvette all at the same protein concentration adjusted to 0.5 mg·mL^−1^. The spectra were recorded on a Shimadzu UV‐2401 PC spectrophotometer (Shimadzu, Kyoto, Japan). The low‐temperature ^57^Fe Mössbauer spectra of the samples were measured as described previously 8 and processed using the MossWinn software program 52. The isomer shift values were referred to α‐Fe foil sample at room temperature.

## Author contributions

NS, KT, and JF designed and performed experiments, analyzed data, and wrote the manuscript. HU conducted structure modeling. JP and PN measured Mössbauer spectra, analyzed data, and drew simulated spectra. All authors revised the manuscript.

## Supporting information


**Table S1.** PCR primers used for construction of PqqE mutant enzymes
