# Supplementary material for: Characterization of auxiliary iron–sulfur clusters in a radical S‐adenosylmethionine enzyme PqqE from Methylobacterium extorquens AM1
Source: FEBS Open Bio. 2017 Oct 18;7(12):1864–79. doi: 10.1002/2211-5463.12314 (PMC5715301; doi:10.1002/2211-5463.12314)
Supplement: Supplementary file 1 — Table S1. PCR primers used for construction of PqqE mutant enzymes [file FEB4-7-1864-s001.docx]

**Table S1.** PCR primers used for construction of PqqE mutant enzymes

| ***Primer*** | ***Sequences*** |
| --- | --- |
| C268S_F | 5′-GCAAGGTGCTGCCCAGCCACGCCGCAGA-3′ |
| C268S_R | 5′-CCTGCGGCGTCACGTTCATCAGCT-3′ |
| C313S_F | 5′-AGCCCTGCCGCTCCAGCGACCGGCGCGA-3′ |
| C313S_R | 5′-CCTTCATCCAGGACGTGCCGCGAT-3′ |
| C310S_F | 5′-CGTCCTGGATGAAGGAGCCCAGCCGCTCCTGCGACCGGCGC-3′ |
| C310S_R | 5′-GCGCCGGTCGCAGGAGCGGCTGGGCTCCTTCATCCAGGACG-3′ |
| C310S/C313S_F | 5′-CGTCCTGGATGAAGGAGCCCAGCCGCTCCAGCGACCGGCGC-3′ |
| C310S/C313S_R | 5′-GCGCCGGTCGCTGGAGCGGCTGGGCTCCTTCATCCAGGACG-3′ |
| D319S_F | 5′-CCTGCGACCGGCGCGAGAAGAGTTGGGGCGGGTGCCGCTGC-3′ |
| D319S_R | 5′-GCAGCGGCACCCGCCCCAACTCTTCTCGCGCCGGTCGCAGG-3′ |
| C323S_F | 5′-GCGAGAAGGATTGGGGCGGGAGCCGCTGCCAGGCGCTGGCG-3′ |
| C323S_R | 5′-CGCCAGCGCCTGGCAGCGGCTCCCGCCCCAATCCTTCTCGC-3′ |
| C28S/C32S_F | 5′-GTATGGGCTGCGCAGCGGGCTGCGGTGCGTCAGCTCGGCG-3′ |
| C28S/C32S_R | 5′-CGCCGAGCTGACGCACCGCAGCCCGCTGCGCAGCCCATAC-3′ |
| C341S_F | 5′-CGCGGCCAACACCGATCCGGCCAGCTCCCTTTCGCCGCTGCACGC-3′ |
| C341S_R | 5′-GCGTGCAGCGGCGAAAGGGAGCTGGCCGGATCGGTGTTGGCCGCG-3′ |
